# Associations between Water Quality Measures and Chronic Kidney Disease Prevalence in Taiwan

**DOI:** 10.3390/ijerph15122726

**Published:** 2018-12-03

**Authors:** Kuan Y. Chang, I-Wen Wu, Bo-Ruei Huang, Jih-Gau Juang, Jia-Chyi Wu, Su-Wei Chang, Chung Cheng Chang

**Affiliations:** 1 Department of Computer Science and Engineering, National Taiwan Ocean University, Keelung 202, Taiwan; kchang@ntou.edu.tw (K.Y.C.); rrayhhuang@gmail.com (B.-R.H.); 2 Division of Nephrology, Keelung Chang Gung Memorial Hospital, Keelung 204, Taiwan; fliawu@yahoo.com; 3 Department of Communications, Navigation and Control Engineering, National Taiwan Ocean University, Keelung 202, Taiwan; jgjuang@mail.ntou.edu.tw (J.-G.J.); jcwu@mail.ntou.edu.tw (J.-C.W.); 4 Clinical Informatics and Medical Statistics Research Center, College of Medicine, Chang Gung University, Taoyuan 333, Taiwan; shwchang@mail.cgu.edu.tw; 5 Division of Allergy, Asthma, and Rheumatology, Department of Pediatrics, Chang Gung Memorial Hospital at Linkou, Taoyuan 333, Taiwan; 6 Department of Electrical Engineering, National Taiwan Ocean University, Keelung 202, Taiwan

**Keywords:** chronic kidney disease, end-stage renal disease, water contaminants, zinc, ammonia, chemical oxygen demand, dissolved oxygen, arsenic

## Abstract

To determine the relationships between exposure to environmental contaminants in water and chronic kidney disease (CKD), we investigated the associations of 61 water attributes with the prevalence of CKD and End-Stage Renal Disease (ESRD) using data from 2005 to 2011 from all 22 counties and cities in the main island of Taiwan. We acquired patient information from the Taiwan Longitudinal Health Insurance Database to calculate the age-standardized CKD and ESRD prevalence rates and linked the patients’ residences to the water quality monitoring data, which were sampled periodically for a total of over 45,000 observations obtained from the Taiwan Environmental Water Quality Information Database. The association analysis adjusting for gender, age, and annual effects showed that the zinc (Zn), ammonia, chemical oxygen demand (COD), and dissolved oxygen in rivers were weakly correlated with CKD (τ = 0.268/0.250/0.238/−0.267, *p* = 6.01×10^−6^/2.52×10^−5^/6.05×10^−5^/3.30×10^−5^, respectively), but none for ESRD. The importances of Zn and COD in rivers were also demonstrated in a CKD regression model. Moreover, an unusually high CKD prevalence was related to arsenic contamination in groundwater. A further prospective cohort study would improve our understanding of what level of environmental water with risky properties could affect the development of CKD.

## 1. Introduction

Understanding risk factors is key to preventing and controlling the development of chronic kidney disease (CKD). The known risk factors for developing CKD can be put into four categories. First, demographic conditions. People at risk are, for example, female [1], over 75 years old [2], non-Hispanic blacks [3], receiving only primary education or no education [4,5], and having a family history of chronic renal diseases [6]. Second, comorbidities include diabetes [1,7], hypertension [1,8], metabolic syndrome [9], heart failure [10], hepatitis B [11], hepatitis C [12], glomerulonephritis [13], hyperuricemia [14], hyperlipidemia [1], anemia [15], and systemic lupus erythematosus [16]. Third, lifestyles, including smoking for more than five pack-years [17], drinking alcohol heavily with 30 g/day [18], betel nut chewing [19], exercising lightly with <30 min of bicycling per day or an equal amount of activities [20], and having a low water intake of <2.0 L/day [21]. The last category is environmental and physiological conditions, including having cadmium in one’s blood and/or urine [22], lead in the blood [23], and organophosphorus herbicides in water [24]. A recent study even suggested that dehydration resulted from climate change may also be a risk factor for developing CKD [25]. Among all of these factors, diabetes and hypertension are highly recognized to associated with CKD [8,26].

Taiwan, infamous for the highest prevalence rate of end-stage renal disease (ESRD) in the world [27,28], has suffered the prevalence of CKD. CKD is also a global health problem. Approximately 13.4% of the world’s population has CKD [29]. The CKD prevalence rate in Taiwan was approximately 11.9%, estimated by a large-scale study of 462,293 Taiwanese adults in 2006 [27]. In other words, approximately 2.03 million Taiwanese people had CKD [27]. CKD, which can be divided into five stages of increasing severity, is becoming an increasingly serious health problem worldwide. Until 2015, Taiwan had the highest prevalence of ESRD in the world (3317 cases per million people) [28]. A total of 77,920 Taiwanese patients received an ESRD diagnosis in 2015 [28]. ESRD is not only life-threatening and inconvenient for patients’ daily living, but also a burden on the society’s medical resources. Therefore, the early diagnosis and prevention of CKD are critical objectives in maintaining a healthy public.

Unprocessed raw water can affect one’s health. Not only can unprocessed water be a source of drinking water, but it can also influence agricultural soils, plants, and animals, subsequently affecting our health. In Taiwan, three categories of water resources (reservoirs, rivers, and groundwater) dominate the sources of drinking water. About three-fourths drinking water is tap water [30]. More than half of tap water comes from reservoirs, about one third from surface water like rivers, and about ten percent from groundwater [31]. Raw water before drinking undergoes a series of water treatment processes, which involve physical, chemical, and biological processes in order to remove contaminants such as pesticides, organic pollutants, and volatile organics. Yet, the remaining contaminants may still be delivered into human bodies.

Although water is essential for kidney function, a full-scale investigation into the environment’s exposure to water contaminants in the development of CKD/ERSD has yet to be conducted in Taiwan in order to identify any potential water-related risk factors for CKD/ESRD. This study aimed to investigate the associations between the 61 water attributes of the three water resources and the CKD/ESRD prevalence rates in Taiwan from 2005 to 2011. To our knowledge, this is the first study to assess the relationships between CKD/ESRD and a wide range of water properties in the environment.

## 2. Materials and Methods

To evaluate the relationships between large-scale water attributes and CKD/ESRD, an ecological study using longitudinal data was performed. The examined longitudinal data involved aggregated de-identified health records and water monitoring data in Taiwanese counties and cities from 2005 to 2011. The unit of observation was either a county or a city in a specific year. To detect a significant relationship, both the annual average water quality values and age-standardized CKD/ESRD prevalence rates of the subjects were utilized. An annual average water quality was derived from the data from all monitoring stations within a particular county or city in a year, where each sample was given an equal weight. Finally, to lower the Type I error, the significances of the 61 relationships were determined using partial correlation with a Bonferroni–Holm correction [32] at an α level of 0.01.

### 2.1. Study Area

**Figure 1:**
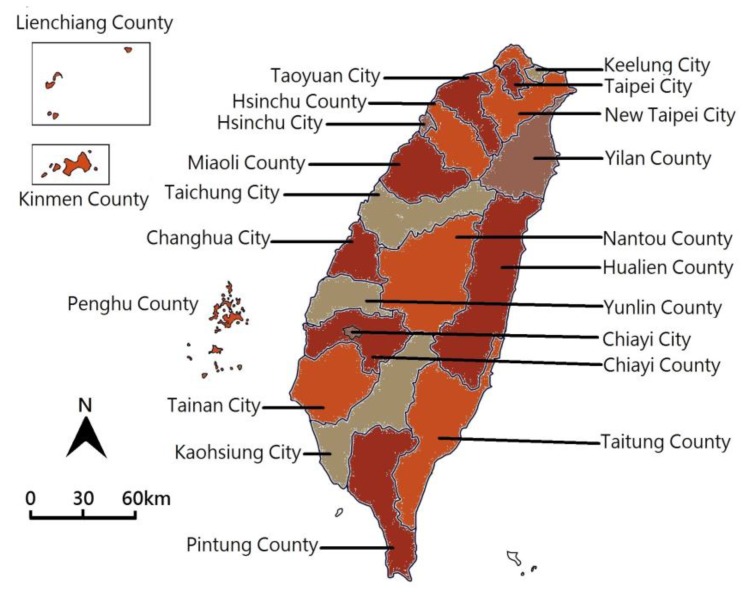
The 22 counties and cities in Taiwan. The map colors have no special meanings.

Taiwan is a narrow and long island with a north-to-south orientation. The total area of the island is approximately 36,193 km^2^. It is located southeast of the Asian continent and is part of the East Asian island arcs in the west bank of the Pacific Ocean. Located in the subtropical climate zone (23° N, 120° E), Taiwan neighbors the archipelagos of Japan and Ryukyu to the north, the Philippines to the south, and mainland China to the west. The two-thirds island area is dominated by rugged mountains that are covered by forests and the rest of the island consists of rolling hills, plateaus, coastal plains, and basins. There are a total of 22 counties and cities in Taiwan (Figure 1).

### 2.2. Water-Quality Monitoring Data

The water-monitoring data were acquired from the Environmental Water Quality Information Database maintained by the Environmental Protection Administration of Taiwan [33]. The monitoring data are divided into five categories according to the source of water resources: reservoirs, groundwater, rivers, beaches, and coastal oceans. Because water resources in Taiwan are distributed unevenly, the number of monitoring stations and observations in each county or city vary. Of all the 1096 water monitoring stations in Taiwan, 448 stations were for groundwater, 319 for rivers, 121 for reservoirs, 105 for coastal oceans, and 103 for beaches. Between 2005 and 2011, these stations collected a total of 51,037 water-monitoring observations, comprising 9345 observations of groundwater, 31,791 of rivers, 4710 of reservoirs, 2662 of oceans, and 2529 of beaches. We selected groundwater, rivers, and reservoirs to be examined because they are closely related to tap water.

There were 61 water quality measures that we examined: 23 for groundwater, 15 for reservoirs, and 23 for rivers. Each water resource monitored a different subset of 36 water quality items. A total of 20 of the 36 monitored items were specific metal or inorganic substances in water and the rest (16 items) belonged to the physicochemical and biological properties of water. Tables S1–S3 for groundwater, reservoirs, and rivers show the levels of water attributes in the study area. 

### 2.3. CKD/ESRD Prevalence Rates

The age-adjusted standardized CKD/ESRD prevalence rates standardized by the World Health Organization (WHO) 2000–2025 standard population were used. This study adhered to the Declaration of Helsinki and was approved by the ethics committee of the Institutional Review Board of Chang Gung Memorial Hospital (IRB No. 100-4385A3 and 102-2508B). This study employed de-identified secondary data from the National Health Insurance Database (NHID) and thus was exempted from informed consent. The original prevalence data were retrieved from NHID, which covered almost 99% of the entire population of Taiwan [34]. The prevalent CKD patients were defined by the presence of the several diagnostic codes (ICD-9-CM codes: 250.4, 274.1, 283.11, 403.1, 404.2, 404.3, 440.1, 442.1, 447.3, 572.3, 580–588, 593, 642.1, 646.2, and 753.1) in at least three ambulatory claims or one inpatient claim between 2005 and 2011. The prevalent ESRD patients were identified if the patient had both an ICD-9-CM code of 585 and an inclusion in the Registry for Catastrophic Illness [1,35,36,37], a rigorous requisite for the NHI payment of dialysis therapies. The details of ICD-9 codes for CKD and ESRD are listed at Table A1 and Table A2. Those excluded were patients who died, who lacked IDs, who were less than 18 years old, and those who exited the insurance program or underwent renal transplantation. The township of cases was identified by the location of the medical setting of the patient with CKD. Briefly, the standardized prevalence rates were age-adjusted to the WHO 2000–2025 standard population using 18 age groups (0–4, 5–9, …, 80–84, 85+). The standardized CKD/ESRD prevalence rates of this study are consistent with those of previous studies [38,39,40].

Like the prevalence rates, the gender ratios—one of the controlling factors—were also standardized with age adjustment. The other demographic variables including the national population estimates, the townships’ population densities, and the townships’ population median ages were obtained from Taiwan’s socio-economic database (http://segis.moi.gov.tw/STAT/). Out of the 25.56 million individuals enrolled in NHID, about 1,000,000 enrolled beneficiaries were randomly sampled in 2005 and were followed up with until 2011. Table 1 describes the baseline characteristics of the study population from 2005 to 2011.

### 2.4. Correlation between Water Quality and CKD/ESRD

To assess the relationship between the average water quality and the CKD/ESRD prevalence rates, partial Kendall correlations [41] were applied. Partial correlation *τ*_yx∙z_ calculates the correlation between *X* and *Y* by controlling *Z* or, more literally, by removing the effects of *Z*. Its formula is as follows:

(1) $$\tau_{\mathrm{yx}}\cdot_{z}\;=\;\frac{\tau_{yx}-(\tau_{yz})(\tau_{xz})}{\sqrt{1-\tau_{yz}^{2}}\sqrt{1-\tau_{xz}^{2}}}$$

where *τ*_yx_, *τ*_zx_, and *τ*_yz_ are Kendall’s correlations [42] between *Y* and *X*, *Z* and *X*, and *Y* and *Z*, respectively. The correlation magnitude was classified into four levels: strong (1.0 ≥ *τ* ≥ 0.7), moderate (0.7 > *τ* ≥ 0.4), weak (0.4 > *τ* ≥ 0.1), and negligible (0.1 > *τ* ≥ 0). When the difference between *τ*_yx∙z_ and *τ*_yx_ was negligible, *Z* had no effect on the relationship between *Y* and *X*, suggesting that *Y* and *X* may have a direct relationship. When *τ*_yx∙z_ < *τ*_yx_, it meant that *Y* and *X* may have a spurious relationship or that *Z* may be a common cause or an intermediate factor between *Y* and *X*. When *τ*_yx∙z_ > *τ*_yx_, the suggestion is that the effect of *X* on *Y* might be independent of that of *Z* on *Y*. In other words, *X* and *Z* have no correlation but, by removing the covariates between *Y* and *Z*, the correlation between *Y* and *X* improves. In our case, we aimed to control the confounding factors such as gender, age, annul, or time-dependent bias. That is, *X*, *Y*, and *Z* are water quality, the prevalence of CKD/ESRD, and control variables (population gender ratio, population median age, and sampling year), respectively. The “ppcor” package in R was used to perform the partial Kendall’s correlation.

### 2.5 CKD Regression Analysis Using Selected Water Attributes

We built a CKD prevalence prediction model using nonparametric generalized additive models (GAMs) [43]. GAMs were used to associate the regional CKD prevalence rates as the dependent variable and the corresponding regional water attributes as the independent variables. In this study, multiple GAMs were constructed and the optimal model was determined using the Akaike Information Criterion [44]. A GAM adjusting for confounding sex, age, and year can be expressed as follows:

(2) $$E\left(Y\right)=B_{0}+s\left(Sex\right)+s\left(Age\right)+s\left(Year\right)+s_{1}\left(X_{1}\right)+s_{2}\left(X_{2}\right)+\cdots +s_{n}\left(X_{n}\right)$$

where *E*(*Y*) refers to the expected regional CKD prevalence rate during the year, *s*( ) are the smoothing functions by splines, and *X_i_* are the selected river quality attributes such as Zn, COD, DO, and NH_3_. The “mgcv” package in R was applied to build the GAMs.

## 3. Results

### 3.1. Associations between Water Attributes and CKD in Taiwan

Table 2 presents the partial correlations adjusted for region-wise population, gender, age, and time-dependent annual effects between the water attributes and the CKD prevalence rates. Only four out of the 61 water attributes that passed the Bonferroni–Holm test displayed significant associations: the CKD prevalence rate was significantly correlated with the magnitude of Zn, ammonia (NH_3_-N), and the chemical oxygen demand (COD) of rivers (*τ* = 0.268/0.250/0.238, *p* = 6.01×10^−6^/2.52×10^−5^/6.05× 10^−5^, respectively) and it was inversely correlated with the magnitude of the dissolved oxygen (DO) of rivers (*τ* = −0.267, *p* = 3.30 × 10^−5^). The results indicated that a region with a higher CKD prevalence rate may be associated with a larger amount of Zn, ammonia, or COD of rivers, as well as a lower magnitude of DO of rivers. 

### 3.2. Associations between Water Attributes and ESRD in Taiwan

Table 3 presents the partial correlations adjusted for region-wise population, gender, age, and time-dependent annual effects between the water attributes and the ESRD prevalence rates. None of the 61 water attributes displayed a significant association with the ESRD prevalence rates because they all failed the Bonferroni–Holm correction test.

### 3.3. Influential Observations Found in the Scatter Plots

**Figure 2:**
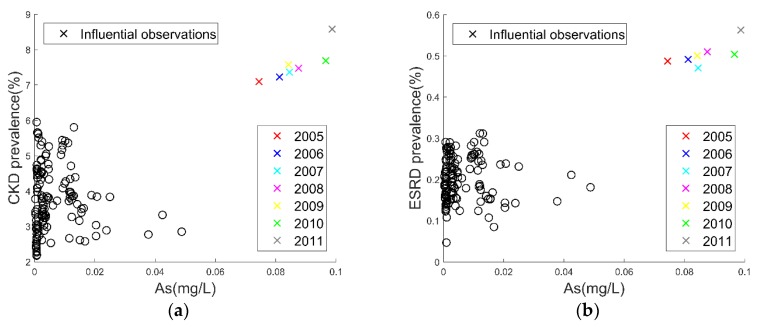
The relationship between arsenic in groundwater and age-standardized chronic kidney disease (CKD)/ end-stage renal disease (ESRD) prevalence rates (2005–2011): (**a**) the CKD prevalence rate in groundwater and (**b**) the ESRD prevalence rate in groundwater. The influential observations are labeled by the color-coded year symbols ‘×’.

Figure 2 illustrates the influential observations between arsenic in groundwater and CKD/ESRD. Seven highly influential observations that were far away from the other observations on the X axis were identified on the top right corner of Figure 2. We realized that all these high influencers came from the same region, Chiayi city. A clear trend was revealed in that the arsenic in the groundwater accompanying the CKD/ESRD prevalence in Chiayi city increased with time. The *Z*-score bar chart of all the monitored substances of Chiayi city (Figure S1) indicates that Chiayi city was like the other places in Taiwan except that it had an unusual high level of arsenic in the groundwater, with a *Z*-score was 3.85. In other words, the concentration of arsenic in the groundwater in Chiayi city was over 75 µg/L, a value that was exceptionally higher than those of the other regions in Taiwan.

We performed an additional analysis by excluding Chiayi city because we suspected that the arsenic in the groundwater of Chiayi city could dominate the association of the CKD/ESRD prevalence. However, albeit with some differences, the associations were rather stable.

### 3.4 Optimal CKD Regression Model Using Selected River Attributes

Table 4 indicates that the GAM built by Zn and the COD of rivers with confounding covariates (sex, age, and year) was the optimal model with the minimum AIC value (−987.6). The larger model with one additional parameter (NH_3_) and the smaller model that considered only Zn had similar AIC performance values to the optimal model, differing by AIC values of 1.3 and 2.4, respectively.

## 4. Discussion

To our knowledge, this is the first ecological study with seven years of monitoring to associate the CKD and ESRD prevalence rates with a wide range of water attributes of the environment. We found that Zn, ammonia, COD, and DO of rivers were weakly correlated with the CKD prevalence rates, but none of them were linked to ESRD under the stringent Bonferroni–Holm test.

The GAM with the Zn and COD of rivers was the best model for the given data (Table 4). Although including additional NH_3_ or omitting COD in the model led to a similar AIC performance when compared to the best model, the results did not support the inclusion of one more parameter (NH_3_). However, the necessity of the inclusion of COD in the model was unclear. The differences in AIC did not support the notion that the Zn only model was substantially inferior to the best model.

Zn in the environment has been linked to CKD. In fact, both our correlation and regression results supported that Zn was a relatively stronger indicator of CKD prevalence than the three other river attributes. A recent study that indicated that Zn in residential soil was a risk factor for CKD progression also supports our findings [45]. We suspect that a Zn contaminated river, which can pollute soil, may reach CKD patients in the same way that Zn contaminated soil does. On the other hand, Zn homeostasis is important for kidney function. A Zn deficiency is present in CKD patients [46]. Although oxidized Zn particles which interfere with Zn homeostasis in rats are toxic [47], further investigations are needed to confirm whether exposure to environmental Zn could cause the development of CKD in humans.

To our knowledge, this is also the first report to link environmental water quality indicators to CKD in humans. Ammonia, COD, and DO all are indicative measures of water quality. They are interrelated in some way. For example, ammonia can acidify water, consume oxygen, and raise the COD. Ammonia can be a key indicator to determine whether water resources are undergoing anthropogenic pollution. Excessive anthropogenic pollutants—which mainly come from unprocessed livestock wastewater, domestic sewage, and industrial manufacturing—are enriched with ammonia. Elevated levels of ammonia in the water have been demonstrated to degenerate the kidneys of Nile tilapia [48]. However, further studies are needed to verify its role in humans.

Like a high level of ammonia in water, a low DO may mean a poor water quality. A low DO may be caused by overfertilization, causing water plants to overgrow and dead plants to draw a lot of bacteria, which, in turn, depletes the DO level. A river with a lower DO can support less aquatic organisms, implying that the environment’s water is more toxic. A region with a lower DO may mean that its environment is more toxic, thus linking it to CKD patients. Besides, hypoxia, a condition defined by the body being deprived of oxygen, is known to accelerate CKD progression [49]. An adequate oxygen supply is important for the functioning of the kidneys. However, it is unclear whether oxygen-deprived water would reduce the oxygen levels in the kidneys.

A high COD, which indicates more oxidizable pollutants, also suggest that the environment is more toxic in our findings. However, there is a lack of direct evidence that a high COD in water would lead to the development of CKD.

Our results linked arsenic in groundwater to higher CKD prevalence. In fact, the relationship between arsenic and CKD has been extensively studied. Previous animal research on mice and dogs confirmed that exposure to arsenic may damage kidney function [50,51]. A recent systematic review of studies on exposure to arsenic and kidney disease mortality in humans over the past 30 years found evidence that generally supported a positive association between the arsenic and CKD [52]. Our study strengthens their finding through the prevalence rate of kidney disease, not the mortality rate, suggesting that exposure to arsenic in groundwater may relate to the development of CKD/ESRD in Taiwan.

The alarmingly high arsenic concentration in groundwater concurrent with the high prevalence rate of CKD/ESRD in Chiayi city might be the best example to demonstrate a plausible link between exposure to arsenic and CKD/ESRD. Low-dose arsenic levels did not exhibit a clear correlation, suggesting that arsenic in groundwater may need to pass a certain threshold to be associated with CKD/ESRD. Because arsenic exists in the natural environment, such as in the soil, air, water, and food, it may enter human bodies through breathing, drinking, or eating. Chiayi is infamous for its 1950s Blackfoot disease endemic [53], which resulted from the exposure to arsenic in drinking water from artesian wells, consequently impairing the blood vessels of the lower limbs of patients and causing them to develop gangrene. Based on the 1962 groundwater data, two ecological studies linked arsenic to the increased mortality of kidney disease in Chiayi [54,55]. They also indicated the exposure to arsenic in locals had since ceased [54,55]. Instead, we used contemporary water monitoring data to associate arsenic with the prevalence of kidney disease. The results led us to speculate the possible long latent effects of arsenic or the recurrent arsenic contamination in Chiayi in its drinking water, crops, or farmed seafood. A recent study reported that arsenic in drinking water in Taiwan was linked to the progression of CKD [56]. However, further investigations are needed to verify our speculation.

Because this was a longitudinal study, patients enrolled in this ecological population-based study were aging. Some died and the population declined each year. The mean age of our patients may be older than the national estimate because only those older than 18 years were included in our analysis. Younger patients were excluded from the study to avoid possible confounding effects in renal diseases that are genetic disorders. However, the aging and shrinking populations should not affect our results as the age factor was controlled and the prevalence rates were applied in our analyses.

The reason why CKD and ESRD did not share the same correlation magnitude with water attributes may either be due to random fluctuations or may involve a complex progression of kidney disease. To determine the reason, more information is required.

### Limitations and Strengths

This study has some limitations. First, this is an observational study at the city or county level so we cannot account for cases smaller than a city or county (e.g., a town or village). Second, although we already adjusted for the gender, age, and annual effects, we still cannot eliminate the possibility that the associations may result from other confounding factors that we did not adjust for. Third, we did not have direct quality measures of drinking water. We instead examined three related water resources. Thus, oral exposure may not necessarily be the same as the environmental exposure studied here. Fourth, unlike incidence cases, prevalence may not capture the full risks of abruptly developing diseases. However, to manifest a renal disease, a long-term exposure may be needed. The exact date at which the exposure started is unknown and to follow up from the first exposure is unfeasible due to the burden of the disease [57,58]. We thus preferred using prevalence data instead of incident cases to measure the burden of the disease during a seven-year time lapse in the Taiwanese population in this ecological study.

However, the use of nationwide health data; the extensive coverage of inorganic, physicochemical, and biological properties of water quality data; and computational analyses adjusted for important covariates, have strengthened the conjecture of this study. Although unmeasured confounders may be still present, we adjusted our data to all the possible covariates whose data were available to us. Needless to say, environmental raw water is important not only because it can be a source of drinking water but also because it is connected to our health through the food chain via agriculture and animals. Although the three water resources were only an approximate to drinking water, they—representing the majority of drinking water sources in Taiwan—were the best ones with a massive amount of information available for this study. Overall, we examined a total of 45,846 water-monitoring observations between 2005 and 2011 to address the issue of the temporality of association. Consequently, the monitored water attributes were found to correlate with the national cohort of patients since 2005 and to 2011 in the development of CKD or ESRD. The seven years of data should be reliable enough to establish meaningful associations in a clinical setting.

In fact, this study has several implications. First, the poor water quality in the environment may be linked to renal disease. Second, regulatory water monitoring of these suspicious contaminants should be strictly done from a public health perspective. Third, water with highly suspicious contaminants should be re-treated or drunk with caution. Fourth, water quality management could be imperative for current public health policymaking. Finally, further clinical trials should be warranted to assure the exact roles of the implicated environmental contaminants, such as Zn in rivers, in renal disease mechanisms.

## 5. Conclusions

Environmental exposure to water containments such as heavy metal Zn is weakly but significantly associated with CKD. Out of the 61 water attributes, only the rivers’ Zn, ammonia, and COD, as well as DO, were found to be weakly correlated with the CKD prevalence rates. Moreover, arsenic contamination in groundwater may be linked to an unusually high CKD prevalence. To confirm our findings, further investigation of individual exposure to the exact amount of water containments should be conducted. Reducing water pollution and better managing the water quality may benefit public health and CKD patients.

## Figures and Tables

**Table 1:** The baseline characteristics of the study population.

Year	2005	2006	2007	2008	2009	2010	2011
Total population, *n*	1,075,535	1,074,803	1,070,511	1,065,938	1,061,195	1,056,549	1,051,921
Age (mean ± SD)	36.4 ± 23.3	37.3 ± 23.4	38.2 ± 23.4	39.2 ± 23.4	40.1 ± 23.5	41.0 ± 23.5	41.9 ± 23.5
Age group							
<40	59.53%	58.30%	56.95%	55.58%	54.20%	52.77%	51.39%
40–49	15.84%	15.94%	16.12%	16.31%	16.42%	16.57%	16.67%
50–59	11.05%	11.89%	12.59%	13.17%	13.75%	14.29%	14.65%
60–69	6.66%	6.61%	6.73%	6.98%	7.30%	7.63%	8.17%
70–79	4.69%	4.80%	4.93%	5.03%	5.15%	5.29%	5.43%
>80	2.24%	2.46%	2.69%	2.93%	3.18%	3.45%	3.69%
Male	48.60%	48.53%	48.45%	48.37%	48.29%	48.21%	48.11%
CKD prevalence	4.00%	4.24%	4.49%	4.73%	4.97%	5.21%	5.53%
ESRD prevalence	0.20%	0.21%	0.23%	0.24%	0.26%	0.27%	0.28%
Comorbidity							
Diabetes	6.40%	7.00%	7.20%	8.20%	8.90%	9.60%	10.30%
Hypertension	11.70%	12.70%	13.80%	14.80%	15.80%	16.80%	17.80%
Hyperlipidemia	7.50%	8.50%	9.61%	10.70%	11.81%	12.90%	14.00%

CKD: chronic kidney disease; ESRD: end-stage renal disease.

**Table 2:** The correlation between water attribute and age-standardized CKD prevalence rate, adjusting for gender, age, and annual effects (2005–2011).

Substance	Groundwater		Reservoir		River	
	*τ*	*p*-Value	*τ*	*p*-Value	*τ*	*p*-Value
**Metal and Inorganics**						
Ag	-		-		−0.088	0.139
As	0.140	0.018	-		0.074	0.213
Ca	0.007	0.902	-		-	
Cd	0.089	0.132	-		−0.038	0.520
Cl	−0.007	0.912	-		-	
Cr	−0.072	0.227	-		−0.009	0.873
Cu	−0.057	0.335	-		0.022	0.714
Fe	−0.091	0.126	-		-	
Hg	-		-		−0.017	0.779
K	0.005	0.933	-		-	
Mg	0.044	0.457	-		-	
Mn	−0.028	0.636	-		0.032	0.594
Na	0.070	0.236	-		-	
NH_3_-N	0.030	0.613	−0.077	0.282	0.250 **	2.52 × 10^−5^ **
NO_2_-N	-		−0.073	0.363	0.211 *	3.79 × 10^−4^ *
NO_3_-N	−0.180	0.002	0.078	0.272	0.191	0.001
Pb	−0.034	0.568	-		0.059	0.318
Se	-		-		−0.061	0.383
${\mathrm{SO}}_{4}^{2-}$	−0.010	0.869	-		-	
Zn	−0.047	0.426	-		0.268 **	6.01 × 10^−6^ **
**Physicochemical and biological properties**						
Alk	0.090	0.128	0.266 *	1.89 × 10^−4^ *	-	
BOD	-		-		0.222 *	1.84 × 10^−4^ *
Chl-A	-		0.021	0.773	-	
COD	-		0.032	0.648	0.238 **	6.05 × 10^−5^ **
Coliform	-		-		0.148	0.013
DO	-		0.031	0.687	−0.267 **	3.30 × 10^−5^ **
EC	0.062	0.292	0.159	0.026	−0.048	0.416
pH	0.125	0.035	0.015	0.833	−0.194	0.001
SD	-		−0.088	0.276	-	
SS	-		0.197	0.006	0.022	0.706
TB	-		0.167	0.019	-	
TDS	0.051	0.389	-		-	
TH	0.019	0.745	0.174	0.014	-	
TKN	-		-		0.235	0.044
TOC	0.031	0.597	0.035	0.627	0.181	0.002
WT	0.130	0.028	0.036	0.618	0.143	0.016

Significance levels of the Bonferroni–Holm test: ** ≤ 0.01, * ≤ 0.05. Abbreviations: Alk: Alkalinity; BOD: Biochemical Oxygen Demand; Chl-A: Chlorophyll-A; COD: Chemical Oxygen Demand; DO: Dissolved Oxygen; EC: Electrical conductivity; SD: Secchi Depth (Transparency); SS: Suspended Solids; TB: Turbidity; TDS: Total Dissolved Solids; TH: Total Hardness; TKN: Total Kjeldahl nitrogen; TOC: Total Organic Carbon; WT: Water Temperature.

**Table 3:** The correlation between water attribute and age-standardized ESRD prevalence rate adjusting for gender, age, and annual effects (2005–2011).

Substance	Groundwater		Reservoir		River	
	*τ*	*p*-Value	*τ*	*p*-Value	*τ*	*p*-Value
**Metal and Inorganics**						
Ag	-		-		−0.034	0.568
As	0.106	0.073	-		−0.017	0.777
Ca	0.007	0.900	-		-	
Cd	0.165	0.005	-		0.021	0.718
Cl	−0.058	0.379	-		-	
Cr	−0.043	0.472	-		−0.009	0.878
Cu	−0.061	0.300	-		0.004	0.949
Fe	−0.079	0.182	-		-	
Hg	-		-		−0.080	0.179
K	−0.112	0.058	-		-	
Mg	−0.097	0.100	-		-	
Mn	−0.016	0.793	-		0.074	0.214
Na	−0.059	0.319	-		-	
NH_3_-N	0.018	0.764	−0.176	0.013	0.094	0.111
NO_2_-N	-		−0.064	0.426	0.109	0.067
NO_3_-N	−0.113	0.057	0.055	0.441	0.086	0.149
Pb	0.030	0.616	-		0.046	0.438
Se	-		-		−0.012	0.867
${\mathrm{SO}}_{4}^{2-}$	−0.103	0.084	-		-	
Zn	−0.135	0.023	-		0.167	0.005
**Physicochemical and biological properties**						
Alk	0.005	0.932	−0.008	0.914	-	
BOD	-		-		0.073	0.215
Chl-A	-		−0.017	0.807	-	
COD	-		−0.056	0.429	0.138	0.020
Coliform	-		-		0.018	0.762
DO	-		0.052	0.501	−0.056	0.388
EC	−0.033	0.577	−0.096	0.178	−0.081	0.172
pH	0.100	0.090	−0.134	0.060	−0.132	0.026
SD	-		0.047	0.560	-	
SS	-		−0.021	0.768	−0.044	0.459
TB	-		0.002	0.973	-	
TDS	−0.021	0.728	-		-	
TH	−0.018	0.766	−0.088	0.218	-	
TKN	-		-		−0.047	0.685
TOC	−0.046	0.439	−0.050	0.486	0.077	0.077
WT	0.010	0.865	0.014	0.845	0.054	0.364

None passed the Bonferroni–Holm test. Abbreviations: Alk: Alkalinity; BOD: Biochemical Oxygen Demand; Chl-A: Chlorophyll-A; COD: Chemical Oxygen Demand; DO: Dissolved Oxygen; EC: Electrical conductivity; SD: Secchi Depth (Transparency); SS: Suspended Solids; TB: Turbidity; TDS: Total Dissolved Solids; TH: Total Hardness; TKN: Total Kjeldahl nitrogen; TOC: Total Organic Carbon; WT: Water Temperature.

**Table 4:** The generalized additive models (GAMs) of CKD prevalence in Taiwan using selected river attributes.

Model	AIC	ΔAIC	R^2^	DE (%)
sex + age + year	−955.0	32.6	0.742	70.7
sex + age + year + COD	−972.2	15.4	0.769	75.0
sex + age + year + DO	−844.2	143.4	0.788	77.2
sex + age + year + NH_3_	−973.0	14.6	0.772	75.6
sex + age + year + Zn	−985.2	2.4	0.821	78.2
sex + age + year + Zn + COD	−987.6	0	0.820	78.8
sex + age + year + Zn + DO	−846.5	141.1	0.805	78.2
sex + age + year + Zn + NH_3_	−983.9	3.7	0.817	78.5
sex + age + year + Zn + COD + NH_3_	−986.3	1.3	0.818	78.9
sex + age + year + Zn + COD + DO + NH_3_	−850.3	137.3	0.809	80.1

ΔAIC_i_ = AIC_i_ − AIC_min_; Abbreviations: AlC: Akaike Information Criterion; COD: Chemical Oxygen Demand; DE: Deviance explained; DO: Dissolved Oxygen.

## Supplementary Materials

Supplementary materials can be found at http://www.mdpi.com/1660-4601/15/12/2726/s1, Figure S1: The *Z*-score bar chart of monitoring water attributes of Chiayi City, Taiwan. Table S1: Descriptive statistics for groundwater in Taiwan. Table S2: Descriptive statistics for reservoirs in Taiwan. Table S3: Descriptive statistics for rivers in Taiwan.

## Abbreviations

The following abbreviations are used in this manuscript:

Alk	Alkalinity
BOD	Biochemical Oxygen Demand
Chl-A	Chlorophyll-A
CKD	Chronic Kidney Disease
COD	Chemical Oxygen Demand
DO	Dissolved Oxygen
EC	Electrical conductivity
ESRD	End Stage Renal Disease
SD	Secchi Depth (Transparency)
SS	Suspended Solids
TB	Turbidity
TDS	Total Dissolved Solids
TH	Total Hardness
TKN	Total Kjeldahl nitrogen
TOC	Total Organic Carbon
WHO	World Health Organization
WT	Water Temperature

## Appendix A

**Table A1:** ICD-9 codes for CKD and ESRD.

Disease	ICD-9 Codes
CKD	250.4, 274.1, 283.11, 403.1, 404.2, 404.3, 440.1, 442.1,
	447.3, 572.3, 580–588, 593, 642.1, 646.2, 753.1
ESRD	585 and associates

**Table A2:** Description of ICD-9 codes.

ICD-9 Codes	Disease Description
250.4	Diabetic Nephropathy
274.1	Gouty Nephropathy
283.11	Hemolytic Uremic Syndrome
403.1	Hypertensive Nephropathy
404.2	Malignant hypertension with renal and cardiac complication
404.2	Malignant hypertension with renal and cardiac failure
440.1	Renovascular atherosclerotic disease
442.1	Aneurysm of renovascular system
447.3	Renovascular hyperplasia
572.3	Renovascular hypertension
580	Acute glomerulonephritis
581	Nephrotic syndrome
582	Chronic glomerulonephritis
583	Nephritis and renal disease
584	Acute kidney failure
585	Chronic kidney failure
586	Kidney failure
587	Renal sclerosis
588	Disease complicated by renal failure
593	Renal and ureter disease
642.1	Gestational, pregnancy related renal disease
646.2	Gestational, pregnancy related renal disease
753.1	Polycystic kidney disease
