# Supplementary material for: Associations between Water Quality Measures and Chronic Kidney Disease Prevalence in Taiwan
Source: Int J Environ Res Public Health. 2018 Dec 3;15(12):2726. doi: 10.3390/ijerph15122726 (PMC6313415; doi:10.3390/ijerph15122726)
Supplement: Supplementary file 1 [file ijerph-15-02726-s001.pdf]

Supplementary materials:

Akaike Information Criterion

To select the optimal model, the Akaike Information Criterion (AIC) is applied. AIC is defined as follows:

$$\text{AIC} = 2\text{K} - 2\log(L(\hat{\theta}|D))$$

where K is the number of parameters in the model and $\log(L(\hat{\theta}|D))$ is the maximized log-likelihood of the model $\hat{\theta}$ given the data *D*. The lower the AIC, the better the model.

Deviance explained (DE)

The deviance D_v of a proposed model is the difference of the log-likelihoods between the proposed model and the perfect model, which is defined as:

$$D_v = 2\log(L(\widehat{\theta_s}|D)) - 2\log(L(\hat{\theta}|D))$$

where $\log(L(\widehat{\theta_s}|D))$ and $\log(L(\hat{\theta}|D))$ are the maximized log-likelihoods of the perfect model $\widehat{\theta_s}$ and the proposed model $\hat{\theta}$, respectively, given the data *D*. Here $\widehat{\theta_s}$ is the saturated model with one parameter for each data point.

Deviance explained (DE) refers to the proportion of the total deviance explained by $\hat{\theta}$.

Detecting the influential data

We applied to Cook's distance D_i to validate the influential observations. Cook's distance D_i of observation *i* is defined as follows:

$$D_i = \frac{\sum_{j=1}^{n}(\bar{y}_j - \bar{y}_{j(i)}\ )^2}{K\frac{1}{n}\sum_{j=1}^{n}(y_j - \bar{y})^2}$$

where $\bar{y}_j$ is the jth fitted value, $\bar{y}_{j(i)}$ is the jth fitted value excluding observation *i*, and K is the number of parameters in the regression model. Those Cook's distances of arsenic in groundwater in Chiayi city were well above three times mean Cook’s distance, suggesting arsenic in groundwater in Chiayi city are influential observations.

Table S1. Descriptive statistics for groundwater in Taiwan

Item	n	Mean±STD	Min	Q1	Median	Q3	Max
**Metal and Inorganics**							
As (mg/L)	9,048	$6.72x10^{-3} \pm 2.34x10^{-2}$	0.0003	0.0004	0.0006	0.0035	0.612
Ca (mg/L)	6,148	87.7 ± 72.7	0.5	39.1	74.9	113.0	630.0
Cd (mg/L)	9,048	$1.05x10^{-3} \pm 4.18x10^{-4}$	0.001	0.001	0.001	0.001	0.014
Cl (mg/L)	7,323	$6.63x10^{2} \pm 3.12x10^{3}$	1.5	12.7	24.3	54.6	40,600
Cr (mg/L)	9,045	$2.08x10^{-3} \pm 2.30x10^{-3}$	0.001	0.001	0.001	0.003	0.161
Cu (mg/L)	9,048	$2.27x10^{-3} \pm 3.45x10^{-3}$	0.001	0.001	0.002	0.003	0.211
Fe (mg/L)	9,048	1.35 ± 4.82	0.005	0.016	0.059	0.662	140
K (mg/L)	6,148	17.3 ± 66.8	0.10	1.80	3.33	7.84	1,160
Mg (mg/L)	6,148	$56.4 \pm 2.05x10^{2}$	0.48	12.3	17.6	30.2	3,880
Mn (mg/L)	9,048	$5.13x10^{-1} \pm 8.76x10^{-1}$	0.005	0.014	0.196	0.675	13.9
Na (mg/L)	6,148	$3.41x10^{2} \pm 1.51x10^{3}$	0.8	16.4	28.8	62.7	17,600
NH_3-N (mg/L)	9,048	1.05 ± 3.44	0.01	0.02	0.07	0.63	123
NO_3-N (mg/L)	9,048	2.25 ± 3.81	0.01	0.03	0.43	3.17	65.4
Pb (mg/L)	9,045	$4.20x10^{-3} \pm 2.48x10^{-3}$	0.003	0.003	0.003	0.005	0.097
SO_4 (mg/L)	9,048	$1.62x10^{2} \pm 4.35x10^{2}$	1.0	41.3	69.1	115	6,510
Zn (mg/L)	9,048	$1.77 x 10^{-2} \pm 4.99x10^{-2}$	0.002	0.002	0.007	0.016	1.410
**Physicochemical and biological properties**							
Alk (mg/L)	6,148	$2.36x10^{2} \pm 1.42x10^{2}$	0	132	218	321	1120
EC (μmho/cm @25℃)	9,048	$2.36x10^{3} \pm 8.08x10^{3}$	58	459	664	1,020	97,300
pH (-)	9,046	6.73 ± 0.58	3.6	6.4	6.8	7.1	10.8
TDS (mg/L)	9,048	$1.64x10^{3} \pm 6.01x10^{3}$	40.5	294	432	668	80,400
TH (mg/L)	9,048	$4.70x10^{2} \pm 1.08x10^{3}$	5.5	159	269	393	17,600
TOC (mg/L)	9,048	1.88 ± 1.89	0.10	0.84	1.43	2.30	71.30
WT (℃)	9,046	26.5 ± 1.7	18.6	25.4	26.6	27.7	34.7

Abbreviations: Alk: Alkalinity; EC: Electrical conductivity; Q1: Lower Quartile; Q3: Upper Quartile; STD: Standard deviation; TDS: Total Dissolved Solids; TH: Total Hardness; TOC: Total Organic Carbon; WT: Water Temperature

Table S2. Descriptive statistics for reservoirs in Taiwan

Item	n	Mean±STD	Min	Q1	Median	Q3	Max
**Inorganics**							
NH_3-N (mg/L)	4,492	$1.08x10^{-1} \pm 4.84x10^{-1}$	0.01	0.01	0.02	0.05	7.35
NO_2-N (mg/L)	2,986	$2.05x10^{-2} \pm 7.80x10^{-2}$	0.001	0.002	0.004	0.008	0.722
NO_3-N (mg/L)	4,448	$4.54x10^{-1} \pm 5.77x10^{-1}$	0.01	0.14	0.34	0.55	8.09
**Physicochemical and biological properties**							
Alk (mg/L)	4,492	94.1 ± 44.2	0.2	70.1	93.7	119	345
Chl_A (µg/L)	4,492	10.3 ± 25.4	0.0	1.2	3.0	8.0	685
COD (mg/L)	4,480	10.5 ± 13.8	4.0	4.0	5.3	10.1	287
DO (ml/min・m2)	3,776	7.75 ± 2.45	0.0	7.0	8.0	9.0	23.7
EC (µmho/cm@25℃)	4,492	$3.67x10^{2} \pm 2.66x10^{2}$	52	228	301	426	4,650
pH (-)	4,492	8.13 ± 0.56	5.2	7.8	8.2	8.4	10.2
SD (m)	2,176	1.62 ± 1.12	0.1	0.8	1.3	2.2	8.2
SS (mg/L)	4,491	8.96 ± 20.5	1.0	2.4	4.3	9.1	702
TB (NTU)	4,492	9.69 ± 25.2	0.3	2.2	4.1	8.9	750
TH (mg/L)	4,492	$1.24x10^{2} \pm 60.2$	13.9	92.9	122	156	783
TOC (mg/L)	4,492	2.68 ± 3.56	0.14	1.09	1.55	2.50	84.7
WT (℃)	4,492	23.5 ± 5.1	6.9	19.7	23.6	27.4	35.4

Abbreviations: Alk: Alkalinity; Chl-A: Chlorophyll-A; COD: Chemical Oxygen Demand; DO: Dissolved Oxygen; EC: Electrical conductivity; Q1: Lower Quartile; Q3: Upper Quartile; SD: Secchi Depth (Transparency); STD: Standard deviation; SS: Suspended Solids; TB: Turbidity; TH: Total Hardness; TOC: Total Organic Carbon; WT: Water Temperature

Table S3. Descriptive statistics for rivers in Taiwan

Item	n	Mean±STD	Min	Q1	Median	Q3	Max
**Metal and Inorganics**							
Ag (mg/L)	7,632	$1.83x10^{-3} \pm 1.13x10^{-3}$	0.001	0.001	0.001	0.003	0.015
As (mg/L)	7,622	$3.57x10^{-3} \pm 4.80x10^{-3}$	0.0003	0.0009	0.0020	0.0043	0.1020
Cd (mg/L)	7,738	$1.03x10^{-3} \pm 9.68x10^{-4}$	0.001	0.001	0.001	0.001	0.067
Cr (mg/L)	7,738	$2.85x10^{-3} \pm 1.34x10^{-2}$	0.002	0.002	0.002	0.003	1.05
Cu (mg/L)	7,632	$2.62x10^{-2} \pm 2.21x10^{-1}$	0.001	0.002	0.003	0.009	13.7
Hg (mg/L)	6,141	$3.22x10^{-4} \pm 1.15x10^{-4}$	0.0003	0.0003	0.0003	0.0003	0.0041
Mn (mg/L)	7,632	$1.76x10^{-1} \pm 4.18x10^{-1}$	0.005	0.030	0.084	0.181	12.2
NH_3-N (mg/L)	22,652	3.54 ± 9.46	0.01	0.05	0.32	2.82	193
NO_2-N (mg/L)	7,738	$1.41x10^{-1} \pm 2.58x10^{-1}$	0.001	0.008	0.044	0.175	8.01
NO_3-N (mg/L)	7,738	1.3 ± 2.15	0.01	0.36	0.74	1.40	56.0
Pb (mg/L)	7,738	$6.70x10^{-3} \pm 1.99x10^{-2}$	0.003	0.003	0.005	0.005	1.28
Se (mg/L)	4,807	$1.02x10^{-3} \pm 1.96x10^{-4}$	0.001	0.001	0.001	0.001	0.006
Zn (mg/L)	7,632	$1.41x10^{-1} \pm 1.95$	0.002	0.010	0.019	0.037	95.7
**Physicochemical and biological properties**							
BOD (mg/L)	22,652	4.86 ± 9.39	1.0	1.0	1.8	5.0	341
COD (mg/L)	22,649	20.7 ± 40.3	4.0	4.5	10.1	24.0	3,180
Coliform (CFU/100mL)	22,652	$4.45x10^{5} \pm 4.99x10^{7}$	10	1,100	11,000	75,000	$6.20x10^{8}$
DO (ml/min・m2)	19,074	6.96 ± 2.76	0.0	5.5	7.7	8.8	23.0
EC (μmho/cm@25℃)	22,652	$2.29x10^{3} \pm 6.89x10^{3}$	31	305	465	751	52,800
pH (-)	22,652	7.83 ± 0.59	2.4	7.5	7.9	8.2	11
SS (mg/L)	22,652	$1.76x10^{2} \pm 2.05x10^{3}$	1.0	8.6	21.1	54.25	241,000
TKN (mg N/L)	1,758	4.71 ± 12.9	0.05	0.32	0.89	3.86	256
TOC (mg/L)	10,556	4.51 ± 7.37	0.10	1.19	2.38	5.15	292
WT (℃)	22,652	24.4 ± 4.8	4.6	20.9	24.6	28.1	37.3

Abbreviations: Biochemical Oxygen Demand; COD: Chemical Oxygen Demand; DO: Dissolved Oxygen; EC: Electrical conductivity; Q1: Lower Quartile; Q3: Upper Quartile; SS: Suspended Solids; STD: Standard deviation; TKN: Total Kjeldahl nitrogen; TOC: Total Organic Carbon; WT: Water Temperature;

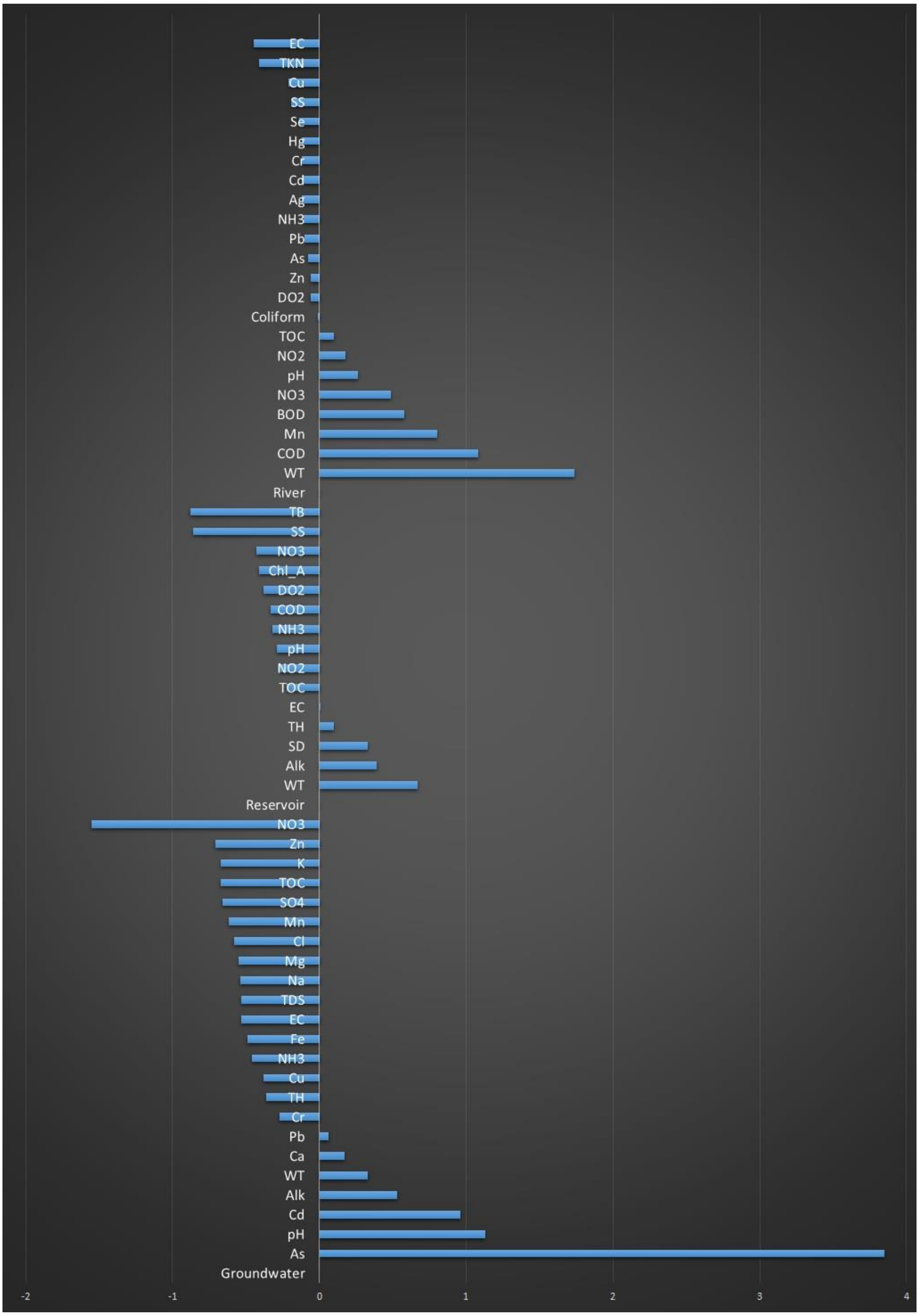

Figure S1. The Z-score bar chart of monitoring water attributes of Chiayi city, Taiwan.
